# Dormant No More: Traumatic Rupture of a Liver Hydatid Cyst Causing Anaphylaxis

**DOI:** 10.7759/cureus.87838

**Published:** 2025-07-13

**Authors:** Hamed Aljanaahi, Omar Farooq Al-Nahhas, Fatima M Alameri, Muneer Al Marzooqi

**Affiliations:** 1 Emergency Medicine, Tawam Hospital, Al Ain, ARE; 2 Clinical Sciences, Ajman University, Ajman, ARE

**Keywords:** anaphylaxis, cyst rupture, echinococcus granulosus, hydatid disease, trauma-induced complication

## Abstract

Hydatid disease, caused by Echinococcus granulosus, commonly affects the liver. Traumatic cyst rupture is rare, and when complicated by anaphylaxis, it poses a life-threatening emergency. A 33-year-old male patient presented with severe abdominal and chest pain after falling from a height. Imaging revealed multiple hepatic hydatid cysts, one of which ruptured. Shortly after the CT scan, the patient developed hypotension, erythema, pruritus, and throat tightness consistent with anaphylaxis. He was treated with epinephrine, corticosteroids, antihistamines, oxygen, and intravenous fluids, stabilized, and admitted for further care. This case illustrates a rare but serious complication of hydatid cyst rupture after trauma. Prompt recognition and interventions are essential, particularly in endemic areas.

## Introduction

Cystic echinococcosis (CE), a zoonotic parasitic disease caused by Echinococcus granulosus sensu lato, remains endemic in many regions, including the United Arab Emirates (UAE) [1,2]. The liver is the most frequently affected organ, accounting for approximately 70% of cases [2]. Although hydatid cysts may remain asymptomatic for years, complications such as rupture can result in severe clinical manifestations, including secondary echinococcosis and anaphylactic shock [3].

Post-traumatic rupture of the hepatic hydatid cysts is a rare but life-threatening emergency. Such rupture may occur spontaneously or following blunt abdominal trauma, leading to the dissemination of hydatid fluid and protoscoleces into the peritoneal cavity. This exposure can trigger severe hypersensitivity reactions, including anaphylaxis, due to the antigenic content of the cysts [1,2]. Clinical presentations can range from mild allergic symptoms to fulminant anaphylactic shock, necessitating rapid diagnosis and urgent management.

Imaging modalities, especially ultrasonography and computed tomography (CT), are essential for identifying hydatid cysts and their complications [3]. In complicated cases, particularly with rupture, surgical intervention is often required. Despite advances in diagnostic and therapeutic strategies, CE continues to present significant clinical challenges, especially in acute settings such as trauma-induced rupture.

## Case presentation

A 33-year-old previously healthy man, with no known allergies, presented to the emergency department with acute abdominal and chest pain after falling from a height of approximately 2-3 meters at his workplace. He denied fever, vomiting, or prior abdominal complaints. On initial evaluation, the patient was alert, oriented, and in visible distress due to pain. His temperature was 37.8°C. He was tachycardic but initially normotensive with normal oxygen saturation on room air.

A trauma-level evaluation was initiated. Focused Assessment with Sonography in Trauma (EFAST) revealed free fluid in the hepatorenal, splenorenal, and pelvic spaces. Cardiac views showed good contractility without evidence of tamponade, and thoracic views ruled out pneumothorax. A trauma pan-CT scan was subsequently performed.

Shortly after the CT scan, the patient developed hypotension (systolic BP in the 90s), raising initial concerns about internal bleeding. Within minutes, he developed acute dyspnea, generalized erythema, pruritus, and throat tightness, requiring supplemental oxygen via face mask at 9 L/min. An anaphylactic reaction, possibly triggered by the IV contrast, was initially suspected.

**Figure 1:**
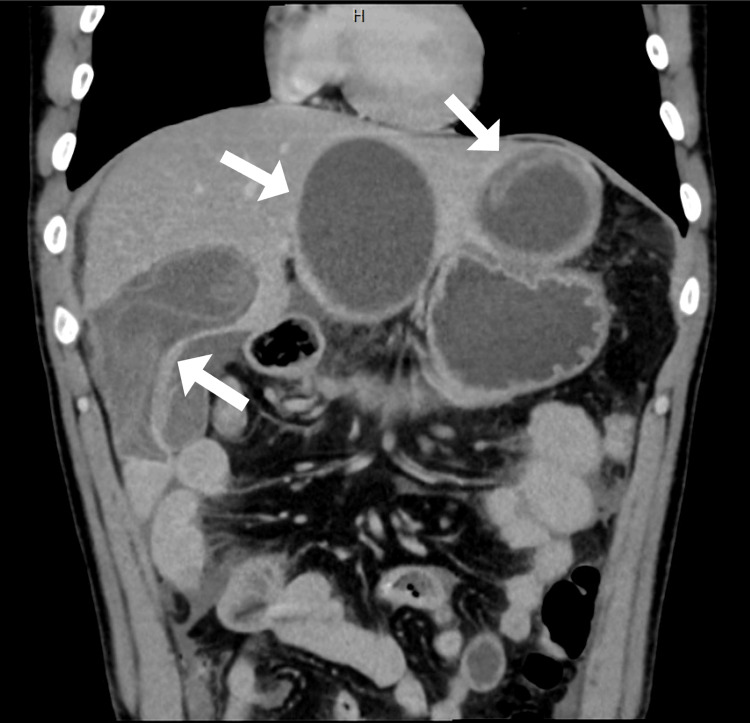
Coronal CT image demonstrating multiple hydatid cysts within the liver

CT findings revealed multiple well-defined cystic lesions in the liver with internal floating membranes, consistent with hydatid cysts (Figure 1).

**Figure 2:**
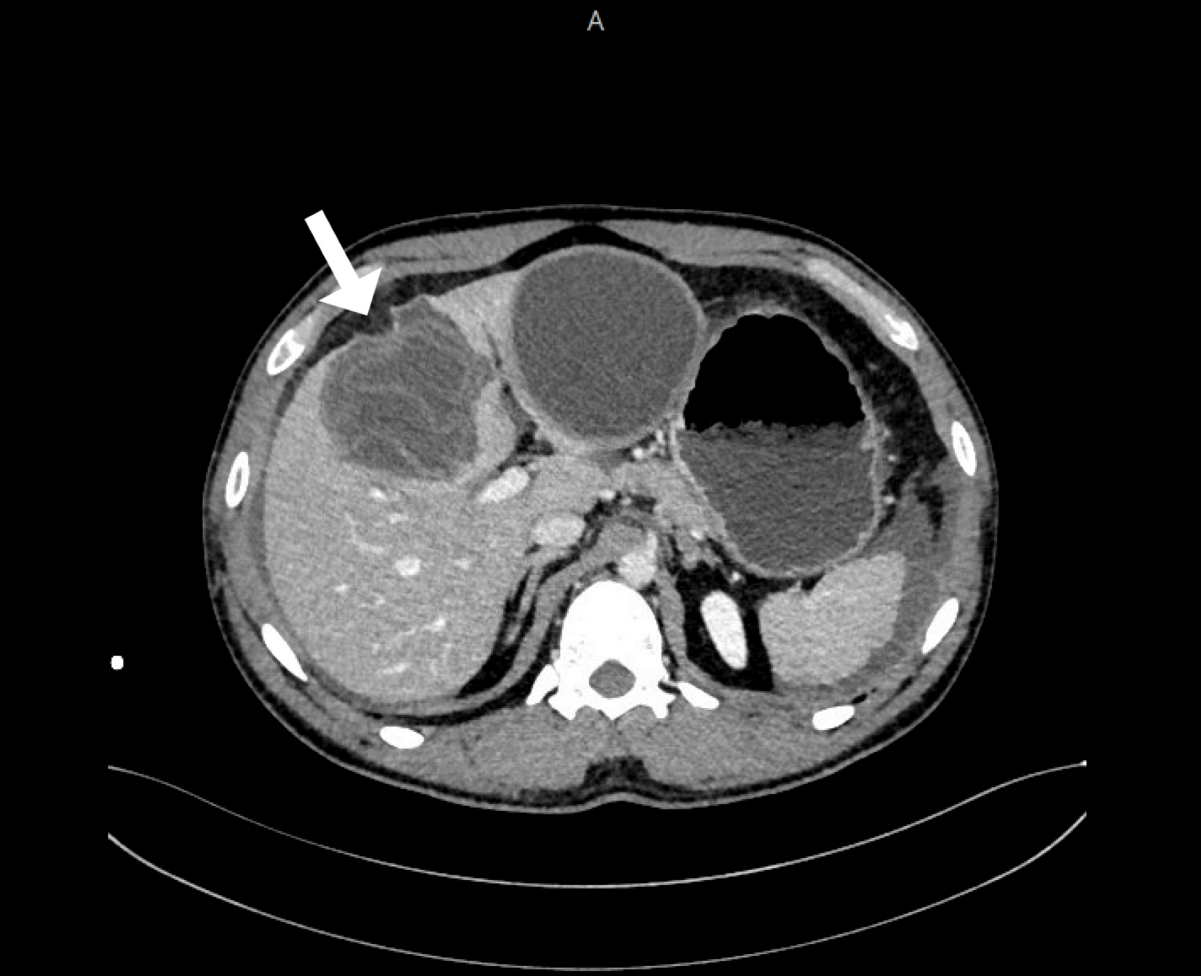
Axial CT image showing multiple hydatid cysts in the liver with one showing signs of rupture (indicated by the arrow)

One cyst demonstrated signs of rupture, with associated mild-to-moderate free fluid in the abdomen and pelvis. No hepatic laceration or other traumatic injuries were identified (Figure 2).

Three potential diagnoses were considered: hemorrhagic shock due to intra-abdominal bleeding, anaphylaxis secondary to hydatid cyst rupture, or contrast-induced hypersensitivity. While EFAST positivity and abdominal fluid suggested trauma, the absence of hepatic laceration and the timing of symptoms aligned more closely with hydatid cyst rupture and associated anaphylaxis. Although contrast reaction remained a possibility, it was considered less likely since the patient had previously tolerated contrast medium. However, we acknowledge that prior uneventful exposure does not definitively exclude sensitization.

Emergency treatment included 0.5 mg intramuscular epinephrine, 125 mg IV methylprednisolone, and 50 mg IV diphenhydramine. The patient received 1 L of IV fluids via pressure bag, high-flow oxygen, 5 mg IV morphine, and paracetamol for pain. Additionally, 2 g IV tranexamic acid was administered. Laboratory investigations showed leukocytosis, a cholestatic liver enzyme pattern with a markedly increased total and direct bilirubin, acute kidney injury, hyperglycemia, and elevated venous lactate (4.0 mmol/L) (Table 1).

**Table 1:** Initial laboratory findings WBC: White blood cell; RBC: Red blood cell; Mean Corpuscular Volume; MCH: Mean Corpuscular Hemoglobin;  MCHC: Mean Corpuscular Hemoglobin Concentration; RDW-CV: Red Cell Distribution Width-Coefficient of Variation; PDW: Platelet Distribution Width; MPV: Mean Platelet Volume; AST: Aspartate Aminotransferase; ALT: Alanine Transaminase; eGFR: estimated Glomerular Filtration Rate; CKD-EPI: Chronic Kidney Disease Epidemiology Collaboration.

Test	Result	Normal range
WBC (×10⁹/L)	14.8	4.0–11.0
RBC (×10¹²/L)	8.46	4.7–6.1 (male)
Hemoglobin (g/L)	161	135–175 (male)
Hematocrit (Hct)	0.5	0.40–0.52 (male)
MCV (fL)	59.3	80–100
MCH (pg)	19	27–33
MCHC (g/L)	321	320–360
Platelets (×10⁹/L)	445	150–400
RDW-CV (%)	19	11.5–14.5
PDW (fL)	12	9–14
MPV (fL)	10	7.5–11.5
Sodium (mmol/L)	137	135–145
Potassium (mmol/L)	4.1	3.5–5.1
Chloride (mmol/L)	103	98–107
CO_2_ (mmol/L)	20	22–29
Anion gap (AGAP; mmol/L)	14	8–16
Creatinine (µmol/L)	125	60–110 (male)
Urea (mmol/L)	6.9	2.5–7.8
Total Bilirubin (µmol/L)	50.7	3–20
Direct Bilirubin (µmol/L)	18.2	0–5
Alkaline Phosphatase (U/L)	146	30–120
AST (U/L)	31	10–40
ALT (U/L)	19	7–56
eGFR (CKD-EPI; mL/min/1.73m²)	65	>60

There was no evidence of hemoperitoneum or solid organ trauma. The general surgery team reviewed the case and, in light of imaging and clinical course, favored the diagnosis of hepatic hydatid cyst rupture as the most probable trigger for the reaction. The patient was stabilized in the emergency department and admitted for observation and further management. He remained hemodynamically stable on oxygen therapy. Surgical deroofing of the cyst was planned, along with initiation of antiparasitic therapy in consultation with the infectious diseases team.

## Discussion

The rupture of hepatic hydatid cysts is an uncommon but serious complication of cystic echinococcosis. When accompanied by anaphylaxis, the condition becomes a medical emergency due to the systemic release of parasitic antigens. While spontaneous cyst rupture has been documented in the literature [4], traumatic rupture is less common and can be diagnostically challenging, particularly in the setting of blunt abdominal trauma.

In this case, the initial clinical impression favored hemorrhagic shock due to positive EFAST findings and hypotension. However, the subsequent development of erythema, pruritus, throat tightness, and dyspnea shifted the diagnostic focus toward anaphylaxis. CT imaging played a pivotal role in identifying the ruptured hydatid cyst and ruling out other causes of the intra-abdominal free fluid.

Similar cases have been reported. Dörterler et al. described spontaneous cyst rupture in a pediatric patient with resultant anaphylaxis requiring urgent intervention [4]. Aabdi et al. reported a case of traumatic pulmonary hydatid cyst rupture leading to shock [5]. Changuel et al. highlighted how trauma-induced hepatic cyst rupture may mimic allergic or hemorrhagic conditions in the acute phase [6].

Our case illustrates the critical need to consider hydatid cyst rupture and anaphylaxis in trauma patients from endemic regions. Misdiagnosis as a contrast reaction or hemorrhagic shock may delay definitive care. Prompt imaging, high clinical suspicion, and timely resuscitation are essential to improve outcomes.

The patient received appropriate anaphylaxis management and underwent surgical deroofing the following day. This combined medical-surgical approach aligns with best practice in similar cases [4-7].

While the clinical and imaging findings strongly favored anaphylaxis secondary to cyst rupture, we acknowledge that contrast-induced hypersensitivity and other peri-procedural allergens (e.g., latex) cannot be completely excluded. Prior exposure to contrast without reaction reduces, but does not eliminate, the risk of sensitization. Ideally, further diagnostic clarification such as serum tryptase measurement during the acute episode and subsequent allergy/immunology consultation (including skin prick and intradermal testing) would have helped confirm the etiology [7]. These investigations were not performed in this case, and this remains a limitation.

Despite this diagnostic uncertainty, timely recognition and treatment of anaphylaxis were crucial and contributed to the patient’s favorable outcome.

## Conclusions

This case underscores the importance of recognizing anaphylaxis as a rare but life-threatening complication of hepatic hydatid cyst rupture, especially following trauma. Emergency physicians in endemic areas should maintain a high index of suspicion and promptly initiate both diagnostic imaging and therapeutic interventions.

However, clinicians should remain aware that in acute anaphylaxis, a definitive identification of the trigger may not always be possible. In our case, hydatid cyst rupture was considered the most probable cause based on clinical and radiological findings, but contrast-induced or other peri-procedural allergic reactions could not be completely ruled out without further allergological workup. A multidisciplinary approach involving emergency medicine, surgery, infectious diseases, and allergy/immunology is critical for optimal management and long-term outcomes.
